# Neuronal Antibody Biomarkers for Sydenham’s Chorea Identify a New Group of Children with Chronic Recurrent Episodic Acute Exacerbations of Tic and Obsessive Compulsive Symptoms Following a Streptococcal Infection

**DOI:** 10.1371/journal.pone.0120499

**Published:** 2015-03-20

**Authors:** Harvey S. Singer, Adda Mascaro-Blanco, Kathy Alvarez, Christina Morris-Berry, Ivana Kawikova, Hilla Ben-Pazi, Carol B. Thompson, Syed F. Ali, Edward L. Kaplan, Madeleine W. Cunningham

**Affiliations:** 1 Departments of Neurology and Pediatrics, Johns Hopkins University School of Medicine, Baltimore, Maryland, United States of America; 2 Departments of Microbiology and Immunology, University of Oklahoma Health Sciences Center, Oklahoma City, Oklahoma, United States of America; 3 Department of Immunobiology, Yale University School of Medicine, New Haven, Connecticut, United States of America; 4 Neuropediatric Unit, Shaare Zedek Medical Center, Jerusalem, Israel; 5 Biostatistics Center, Johns Hopkins Bloomberg School of Public Health, Baltimore, Maryland, United States of America; 6 Department of Pediatrics, University of Minnesota Medical School, Minneapolis, Minnesota, United States of America; Medical University of Innsbruck, AUSTRIA

## Abstract

Several autoantibodies (anti-dopamine 1 (D1R) and 2 (D2R) receptors, anti-tubulin, anti-lysoganglioside-GM1) and antibody-mediated activation of calcium calmodulin dependent protein kinase II (CaMKII) signaling activity are elevated in children with Sydenham’s chorea (SC). Recognizing proposed clinical and autoimmune similarities between SC and PANDAS (pediatric autoimmune neuropsychiatric disorder associated with a streptococcal infection), we sought to identify serial biomarker changes in a slightly different population. Antineuronal antibodies were measured in eight children (mean 11.3 years) with chronic, dramatic, recurrent tics and obsessive-compulsive disorder (OCD) associated with a group A β-hemolytic streptococcal (GABHS) respiratory tract infection, but differing because they lacked choreiform movements. Longitudinal serum samples in most subjects included two pre-exacerbation samples, Exac), one midst Exac (abrupt recurrence of tic/OCD; temporally association with a GABHS infection in six of eight subjects), and two post-Exac. Controls included four groups of unaffected children (n = 70; mean 10.8 years) obtained at four different institutions and published controls. Clinical exacerbations were not associated with a significant rise in antineuronal antibody titers. CaMKII activation was increased at the GABHS exacerbation point in 5/6 subjects, exceeded combined and published control’s 95th percentile at least once in 7/8 subjects, and median values were elevated at each time point. Anti-tubulin and anti-D2R titers did not differ from published or combined control group’s 95th percentile or median values. Differences in anti-lysoganglioside-GM1 and anti-D1R titers were dependent on the selected control. Variances in antibody titers and CaMKII activation were identified among the institutional control groups. Based on comparisons to published studies, results identify two groups of PANDAS: 1) a cohort, represented by this study, which lacks choreiform movements and elevated antibodies against D2R; 2) the originally reported group with choreiform movements and elevated anti-D2R antibodies, similar to SC. Increased antibody mediated CaMKII activation was found in both groups and requires further study as a potential biomarker.

## Introduction

Sydenham chorea (SC), the neurological manifestation of rheumatic fever, is associated with antibodies against group A β-hemolytic streptococci (GABHS) that cross react with either neuronal extracellular cell surface and/or intracellular (cytoplasmic or cytoskeletal) antigens [1–4]. A similar mechanism has also been proposed for children who develop the acute fulminant onset of movement and behavioral changes, such as tics and OCD, following a streptococcal infection. This latter group known by the acronym PANDAS (pediatric autoimmune neuropsychiatric disorder associated with a streptococcal infection) was first proposed by Swedo and colleagues in 1998 [5]. On a clinical basis concerns have been raised about the defining characteristics and criterion for PANDAS, [6–13], however, studies have not been performed to distinguish whether affected individuals can be differentiated into different groups based on measureable biomarkers. The current study attempts to clarify some surrounding issues by characterizing the presence of antibodies associated with SC in a population of individuals with chronic tics and OCD meeting the criteria for PANDAS, but lacking choreiform (piano-playing) movements during symptom exacerbations [10, 14] **and** possibly having a more “chronic” than relapsing-remitting course [15].

In SC, it is suspected that dopamine (D1 and D2) receptors are the primary antibody target [3, 16], although cross reactive antibodies are also generated which bind to CNS lysoganglioside-GM 1 [17], and the cytoskeletal protein tubulin [18] (See Table 1). Despite the lack of a definitive specific epitope on neuronal cells, the mechanism causing neurological symptomatology is believed to involve the alteration of neuronal cell signal transduction via calcium calmodulin dependent protein kinase II (CaMKII) activation [2, 17, 18]. Supporting data from animal models includes: rats immunized with GABHS developed antibodies against D1 and D2 receptors and clinically showed compulsive–like behaviors [19] and passively-transferred serum obtained from GABHS-immunized mice caused behavioral disturbances [20].

**Table 1:** Anti-neuronal antibody studies in Sydenham Chorea: Anti-D1R and Anti-D2R.

Publication Details	n	Samples	Assay	Result	Comments	
**Anti-D1R**						
Brimberg et al., 2012 [19]	8	SP	ELISA-titer.	Pos	Elevated compared to 19 controls. Extrapolated from table: Mean SC titer = 3,300 and Control = 1,050.	
Ben-Pazi et al., 2013 [16]	22	SP	ELISA-titer.	Pos	Elevated compared to 22 controls; median/mean SC titer = 4,000/7,045 and Control = 2,000/na. Anti-D2R/D1R correlate with clinical symptoms.	
Cox et al., 2013 [3]	10	SP	ELISA-titer.	Pos	Elevated compared to 18 controls. Overlaps with Brimberg's subjects.	
Dale et al., 2012 [21]	30	SP	FlowCyt-CBA.	Neg	No binding to surface D1R antigens transfected in HEK293 cells.	
**Anti-D2R**						
Brimberg, et al., 2012 [19]	8	SP	ELISA-titer.	Pos	Compared to 19 controls; Extrapolated from table: Mean SC titer = 14,375 and Control = 6,250. SC reacted significantly more against D2R than D1R.	
Ben-Pazi et al., 2013 [16]	22	SP	ELISA-titer.	Pos	Compared to 22 controls; median/mean SC titer = 16,000/20,636 and Control = 8,000/na. Anti-D2R/D1R correlate with clinical symptoms.	
Cox et al., 2013 [3]	10	SP	ELISA-titer.	Pos	Compared to 18 controls; Mean SC = 17,600 and Control = 6000. Overlaps with Brimberg's subjects. D2R epitope identified.	
Dale et al., 2012 [21]	30	SP	FlowCyt-CBA.	Pos	Reactive in 10/30 SC patients; binding to surface D2R long antigens transfected in HEK293 cells.	
Cox et al., 2013 [3]	4	SP	FlowCyt-CBA.	Pos	Reactive in 4/4 SC patients (acute vs convalescent); binding to FLAG epitope tagged D2R long antigen transfected in HEK293 cells.	
Cox et al., 2013 [3]	8	SP	cAMP Assay	Pos	D2R signaling assay in human fibroblast L cell line expressing D2R. Positive compared to 7 controls.	
**Anti-Tubulin**					
Kirvan et al., 2007 [18]	1	SP	ELISA-OD	Pos	Acute sera activity greater than convalescent sera and reacted to monoclonal AB 24.3.1 and tubulin.
Ben-Pazi et al., 2013 [16]	22	SP	ELISA-titer	Neg	Not significantly elevated compared to 22 controls (similar to control group 3 in the current manuscript).
**Anti-LysoGM1**					
Kirvan, et al., 2003 [17]	1	SP	ELISA-OD	Pos	Three human monoclonal antibodies and acute chorea sera reacted with lysoganglioside GM1.
Ben-Pazi et al., 2013 [16]	22	SP	ELISA-titer	Neg	Not significantly elevated compared to 22 controls (similar to control group 3 in the current manuscript).
**CaMKII Activation**					
Kirvan et al., 2003 [17]	5	SP	SKNSH cells	Pos	Induction of CaMKII by acute SC sera (n = 5) and human monoclonal AB 24.3.1, but not by SC convalescent sera (n = 3) or control (n = 3).
Kirvan et al, 2006b [22]	6	SP	SKNSH cells	Pos	Activation in acute SC (n = 6, mean = 220% above basal) compared to normal control (n = 5, mean = 110), ADHD (n = 10, m = 104), Tics (n = 10, mean = 90), OCD (n = 6, mean = 85).
Kirvan et al., 2006a [2]	7	SP	SKNSH cells	Pos	Range of activation (% increased from basal): acute SC (n = 7; 195–260%), SC convalescent (n = 5; 105–130%), and healthy controls (n = na, 98–116%).
**Other**					
Brilot et al., 2011 [23]	11	SP	FlowCyt-CBA.	Pos	Binding to SH-SY5Y cells having neuronal and DA characteristics; 11 healthy controls; 11 neurologic controls.

Abbreviations: cAMP (Cyclic adenosine monophosphate); CI (competitive-inhibition); DA (dopamine); D1R (dopamine 1 receptor); D2R (dopamine 2 receptor); FlowCyt-CBA (flow cytometry cell based assay); HEK cell human embryonic kidney cells); L (longitudinal samples); Neg (negative); Pos (positive); SP (single-point-in-time samples), CaMKII (calcium calmodulin dependent protein kinase II), Lyso GM1 (Lysoganglioside GM1), and NA (not available).

Ongoing attempts to confirm an immune-mediated process as the underlying mechanism in PANDAS (see Table 2), tics [24], OCD [25] have been equivocal depending on the study group. Serum antibody reactivity in children against antigens at 60, 45, and 40 kDa in post-mortem basal ganglia (later defined as pyruvate kinase M1, neuron-specific and non-neuronal enolase, and aldolase C) have been reported [26, 27], but could not be duplicated [14, 28]. No correlation was identified between exacerbation of symptoms and changes in anti-neuronal antibodies against caudate, putamen, or frontal cortex (BA 10) [14], and the results of immunofluorescent histochemical studies on brain tissues have been variable [29, 30]. Several reports have suggested that individuals with PANDAS possessing choreiform (“piano-playing”) movements have similar anti-neuronal antibodies to those identified in SC, including anti-D1R, anti-D2R [3, 19], and anti-lysoganglioside-GM1 [17], as well as antibodies that activate CaMKII activity [2, 18, 22] (Table 2). Antibody binding to transfected D1 and D2 receptors in PANDAS has been variable depending on the cell line and clinical presence of choreiform movements [3, 21], and were not increased to differentiated SH-SY5Y cells [23].

**Table 2:** Anti-neuronal antibody studies in PANDAS.

Publication Details	n	SP/L	Assay	Result	Comments
**Anti-D1R**					
Brimberg et al., 2012 [19]	27	SP	ELISA-titer.	Pos	With piano-playing movements. Elevated compared to 19 Controls (overlaps with this study's Group 1). Extrapolated from table: PANDAS mean titer = 2,400 and Control = 1,050. Acute>convalescent.
Cox et al., 2013 [3]	27	SP	ELISA-titer.	Pos	With piano-playing movements. Overlaps with Brimberg's subjects, 18 controls (overlaps with this study's Group 1).
**Current**	6	L	ELISA-titer.	Mixed	No correlation with clinical exacerbation. Individual samples: 3/30 at or above combined control 95th %tile (n = 70; titer = 8,000), whereas 22/30 were at or above the lowest single control group's 95%tile (Group = 1, n = 15, titer = 2,000).
Dale et al., 2012 [21]	22	SP	FlowCyt-CBA.	Neg	No binding to surface D1R antigens transfected in HEK293 cells.
**Anti-D2R**					
Brimberg et al., 2012 [19]	27	SP	ELISA-titer.	Pos	With piano-playing movements. Elevated compared to 19 controls (overlaps with this study's Group 1). Extrapolated from table, PANDAS mean titer = 12,500 and Control = 6,250. Acute>convalescent.
Cox et al., 2013 [3]	27	SP	ELISA-titer.	Pos	With piano-playing movements. Elevated compared to controls. Data for subjects and controls overlaps with Brimberg et al., 2012. PANDAS mean titer 13,449 as compared to Control = 6,000 (n = 18).
**Current**	6	L	ELISA-titer.	Neg	No obvious piano-playing choreiform movements. No correlation with clinical exacerbation. No sample was significantly elevated above the combined control 95th %tile (n = 70; titer = 24,000) or the lowest single control group's 95th %tile (group = 2, n = 17, titer = 9,600).
Morris-Berry et al., 2013 [31]	39	SP/L	ELISA-OD	Neg	Not different from SP controls (n = 15); no correlation with clinical exacerbation (n = 12).
Dale et al., 2012 [21]	22	SP	FlowCyt-CBA.	Neg	No binding to surface D2R antigens transfected in HEK293 cells.
Cox et al., 2013 [3]	4	SP	FlowCyt-CBA.	Neg	With piano-playing movements. No binding to surface D2R antigens transfected in HEK293 cells.
Cox et al., 2013 [3]	7	SP	cAMP Assay	Pos	D2R signaling assay in human fibroblast L cell line expressing D2R. With piano-playing movements. Positive compared to 7 controls.
**Anti-Tubulin**					
Morris-Berry et al., 2013 [31]	40	SP/L	ELISA-OD	Neg	Not differ from SP controls (n = 24); no correlation with clinical exacerbation (n = 12).
**Current**	6	L	ELISA-titer.	Mixed	No correlation with Exac. No sample at or above combined control 95th %tile (n = 70; titer = 3,100), whereas 11/30 were at or above the lowest single control group's 95%tile (group = 2, n = 17, titer = 1,000).
**Anti-LysoGM1**					
Singer et al., 2008 [14]	12	L	ELISA-OD	Neg	No values more than twice blank.
Kirvan et al., 2006b [22]	16	SP	CI-ELISA	Pos	Competitive-inhibition ELISA of serum antibody reactivity to bound N-acetyl-B-D-glucosamine conjugated to bovine serum albumin: significantly higher in PANDAS vs. non-PANDAS sera (P = 0.026).
**Current**	6	L	ELISA-titer.	Mixed	No correlation with Exac. Individual samples: 6/30 were at combined control 95th %tile (n = 69; titer = 1,280), whereas 26/30 were above the lowest single control group's 95%tile (group = 1, n = 14, titer = 216).
**CaMKII Activation**					
Kirvan et al., 2006b [22]	16	SP	SKNSH cells	Pos	PANDAS (n = 16 mean = 145% above basal); acute greater than convalescent. Normal controls (n = 5, mean = 110), ADHD (n = 10, m = 104), Tics (n = 10, mean = 90), OCD (n = 6, mean = 85).
**Current**	8	L	SKNSH cells	Mixed	No correlation with clinical exacerbation. Individual samples: 15/32 were at or above combined control 95th %tile (n = 37; activation value = 157), whereas 31/32 were at or above the lowest single control group's 95%tile (group = 1, n = 15, activation value = 99).
**Binding to SH-SY57 cells**					
Brilot et al., 2011 [23]	12	SP	FlowCyt-CBA.	Neg	Binding to SH-SY5Y cells; 11 healthy and 11 neurologic controls.

Abbreviations: cAMP (Cyclic adenosine monophosphate); CI (competitive-inhibition); DA (dopamine); D1R (dopamine 1 receptor); D2R (dopamine 2 receptor); Exac (Exacerbation); FlowCyt-CBA (flow cytometry cell based assay); HEK cell human embryonic kidney cells); L (longitudinal samples); Neg (negative); Pos (positive); SP/L (single-point-in-time samples longitudinal); SP (single-point-in-time samples), CaMKII (calcium calmodulin dependent protein kinase II), Lyso GM1 (Lysoganglioside GM1), and NA (not available).

In order to further evaluate the possible role of autoantibodies associated with SC in our group of children with chronic recurrent episodes of acute fulminant tics and OCD without choreiform movements, serum antibodies were assayed by ELISA against intracellular (tubulin) and extracellular (lysoganglioside-GM1, D1 and D2 receptors) neuronal markers and CaMKII antibody mediated neuronal signaling activity. This study had 3 goals: 1) prospectively evaluate the longitudinal course of antibody measurements using five serial serum samples that were available at time points approximately 15 and 5 weeks prior to a clinical exacerbation, within two weeks of the exacerbation, and at two intervals approximately 5 and 10 weeks following the exacerbation. We hypothesized that either the anti-neuronal antibodies would increase during a pre-exacerbation period, peak about the time of clinical exacerbation, and then gradually decline or that the anti-neuronal antibodies would be chronic and elevated at all-time points compared to controls. 2) Compare anti-neuronal antibody values in a combined age-matched, multi-institutional derived set of controls. 3) Evaluate the possibility of different anti-neuronal autoantibody profiles in the two reported groups of children with recurrent tics and OCD precipitated and exacerbated by a streptococcal infection meeting the criteria for PANDAS; the originally reported PANDAS group possessing choreiform movements and elevated anti-D2R antibodies and our cohort which lacks choreiform movements.

## Materials and Methods

### Subjects

A subset of prospectively collected serial serum samples was available from eight children, participating in a longitudinal study, who had recurrent episodes of fulminant exacerbations of tics and/or OCD that met published criteria for PANDAS [10] as originally defined by Swedo et al [5]. The cohort, defined below, will herein be labeled as “PANDAS-chronic tics and OCD” to permit separation from the original PANDAS group who presented with choreiform movements (“PANDAS-choreiform”). Diagnostic criteria included: 1) the presence of OCD and/or a chronic tic disorder; 2) age at onset between three years and the beginning of puberty; 3) an episodic clinical course characterized by the abrupt onset of symptoms and a pattern of dramatic recurrent symptom exacerbations and remissions; 4) a temporal relationship between GABHS infection and the clinical course of illness (onset and/or exacerbations). A positive temporal relationship with GABHS required laboratory evidence of an antecedent GABHS infection within nine months of illness onset and/or within four weeks of clinical exacerbation; and 5) neurologic abnormalities, such as motoric hyperactivity or tics being present during a symptom exacerbation. All participating subjects had at least two clinical GABHS-associated clinical exacerbations, although the precise number and timing of prior exacerbations is unknown. No subject had chorea and no obvious fine piano-playing choreiform movements were reported by site investigators [10]. This study and its associated consent form were approved by the individual Institutional Review Boards at Cincinnati Children Hospital, Harvard University/McLean Hospital, Yale University, University of Alabama, New York University, Johns Hopkins University, North Shore-Long Island Jewish Health System, University of Rochester, Washington University, and University of California San Francisco. Written inform consent was provided by parent or guardian and assent provided by the subject.

In our cohort, six children (four male, two female) aged 10.9 ± 2.5 years (mean ± SD) had a clinical exacerbation temporally associated with a GABHS infection (exacerbation with streptococcal infection, ExWS); four “definite” and two “probable.” Two additional children (female aged 11 and male aged 14 years) had a clinical exacerbation having no association with a GABHS infection (exacerbation without streptococcal infection, ExWOS) (Table 3). A “definite” GABHS infection required the presence of three criteria including a pharyngeal Group A streptococcal isolate that was not previously present, a significant rise (0.2 log) in anti-streptolysin O (ASO) and/or anti-DNAse B titer, and clinical signs and symptoms compatible with GABHS infection (fever, sore throat, pharyngeal erythema or exudates) [32]. A “probable” GABHS infection had two of the three previously listed criteria. An exacerbation was declared when the treating neurologist or psychiatrist determined that the subject experienced a dramatic worsening of tics or OCD which lasted for at least 5 days, and was not related to an alteration of prescribed medications. The operational definition of clinical exacerbation was selected to reproduce clinical practice, and was reviewed at annual meetings of the research group [10].

**Table 3:** Clinical data on PANDAS-chronic tics and OCD subjects with an exacerbation associated with a streptococcal infection (ExWS) and on subjects with an exacerbation without an associated streptococcal infection (ExWOS).

ExWS							ExWOS	
Subject ID #	1	2	3	4	5	6	7	8
Gender	F	M	M	M	F	M	F	M
Age at exacerbation (years)	14.1	9.5	9.7	9.7	8.3	14.1	11.3	14.2
Exacerbation								
Definite/probable strep	Definite	Definite	Probable	Probable	Definite	Definite	—	—
Clinical Worsening								
Tics	yes	yes	yes	yes	yes	yes	yes	yes
OCD	no	no	no	yes	yes	yes	yes	no
ADHD	no	no	no	yes	yes	no	no	no

Abbreviations: OCD (obsessive compulsive disorder); ADHD (attention deficit hyperactivity disorder); GABHS (Group A beta hemolytic streptococcal infection).

### Controls

Control sera, from children who were normal, had no tics or OCD, and did not display signs of neurological, autoimmune or acute infectious disease, were provided to the Immunology laboratory at the University of Oklahoma Health Sciences Center (OUHSC). The majority of controls were normal individuals and some were medical disease controls. In order to reduce the possibility of a recent undiagnosed GABHS infection in the control population, all controls with an ASO titer exceeding 400 units were removed from the analysis. The level of 400 was selected as a cutoff based on studies showing that the upper limit of normal varies with age and population [33, 34].

Control sera were obtained from several different institutions: Group 1 (n = 15; mean age 11.4 years, range 7–15.5, ten males, five females) were provided from the National Institute of Mental Health, Bethesda, MD, (Dr. Susan Swedo) and the Yale Child Study Center, New Haven CT, (Dr. James Leckman). This control cohort has been previously published [3, 19]; Group 2 (n = 17; mean age 13.1 years, range 7–17, nine males, eight females) were provided from the Yale Child Study Center by Drs. Ivana Kawikova and James Leckman; Group 3 (n = 17; mean age 10.1 years, range 5–19, seven males, ten females) from the Shaare Zedek Medical Center, Jerusalem, Israel (Dr. Hilla Ben-Pazi). This published group was primarily of Ashkenazi Jewish ethnicity and some had systemic diseases such as Gaucher and spherocytosis [16]; and Group 4 (n = 21; mean age 8.9 years, range 5–15, 11 males, ten females) from the Johns Hopkins Hospital (Dr. Harvey Singer). Some individuals in this latter group were normally developing siblings of children with autism. Samples were re-assayed after being published using a different antibody determining methodology [31].

### Samples

Serum samples were collected aliquoted, frozen, and stored. For the majority of longitudinal analyses, serum samples of each subject were collected at five time points; two prior to the clinical exacerbation point (Exac) (Pre-Exac 1, about 15 weeks before; Pre-Exac 2, about five weeks before), one midst the clinical exacerbation, and two following the exacerbation (Post-Exac 1, about five weeks after; and Post-Exac 2, about ten weeks after). Control and longitudinal subject sera were evaluated in the Immunology laboratory at OUHSC with ELISA titer-based assays for IgG antibodies against tubulin, lysoganglioside-GM1, and dopamine D1 and D2 receptors, as well as for CaMKII antibody-mediated neuronal cell signaling activity above the basal level in a neuronal cell line [17, 19]. Due to insufficient sera, anti-neuronal antibodies were not assayed in the two subjects with an exacerbation without a streptococcal infection and CaMKII activity could not be determined at all-time points in 5/8 subjects. Laboratory personnel were blinded to subject status and sample order.

### Assays

#### ELISA

To determine the levels of SC-associated autoantibodies, titer-based ELISA assays were performed in Immunolon microtiter plates (Fisher Thermo Scientific) on serum samples using purchased tubulin (Purified bovine brain tubulin; MP Biomedicals), lysoganglioside-GM1 (Sigma-Aldrich), dopamine D1 receptor (Perkin Elmer), and dopamine D2 receptor long isoform (Perkin Elmer) and a methodology previously described [18]. Titers represent the minimum serum dilution required to achieve an optical density reading of 0.1 after two hours of incubation.

#### CaMKII activity

To assess whether the serum contains autoantibodies having a functional impact on neuronal cells, CaMKII activity was measured. SKNSH human neuroblastoma cells (purchased from ATCC) were plated at 1x10^7^ overnight under standard cell culture conditions and preincubated with supplemented F12 media for 30 minutes at 37°C, 5% CO_2_. Cells were then cultured for 30 minutes with serum diluted 1/100 and cells were removed as previously described [2, 17, 22]. Cell pellets were solubilized in 0.3 ml of protein extraction buffer with proteinase inhibitors and subjected to homogenization. Enzymatic activity was measured using the CaMKII assay system (Promega, Madison, WI) according to manufacturer's instructions. In brief, 5 μl of cell lysate was incubated with 50μM peptide substrate and ATP^32^ for 2 minutes at 30°C [17]. The sample was spotted onto the capture membrane and washed. Radioactivity retained on the membrane was determined by scintillation counting. The specific activity of the enzyme in pmol/min/μg was determined, and the percentage activity above baseline was calculated for each sample. Both positive and negative sera and a baseline control to measure constitutive enzyme activity were used in the standardization and calculation of the results of the test. CaMKII assay results are reported as percentages above the basal rate (activity obtained in the absence of serum). CaMKII activation has been shown for specific mAb immunoglobulins and to be correlated with serum IgG. Specific blocking with antigen or removal of IgG using anti-IgG beads eliminates the CaM kinase antibody mediated cell signaling activity from sera [17].

#### ASO and anti-DNase B titers

Throat culture processing, streptococcal characterization, M typing and/or emm-gene sequence typing, ASO and anti-DNase B assays for the prospectively followed subjects were performed in the World Health Organization Streptococcal Reference Laboratory at the University of Minnesota Department of Pediatrics using standard neutralization tests. The microbiologic and serologic methods have been previously described [35]. ASO antibody testing for controls was measured by nephelometry in laboratories at each of the contributing institutions.

### Statistical analyses

Statistical analyses were conducted using SPSS 17.0 (SPSS Inc., Chicago, IL) and STATA 11.2 (STATA Corp., College Station, TX) software.

#### Controls

Distributions of the control group data were reviewed. For each antibody and CaMKII activity, the four control groups were compared using the Kruskal-Wallis non-parametric analysis of variance [36], corrected for ties. These tests were followed by multiple comparisons using an experiment-wise error rate of 0.05, as appropriate. Using the combined group of controls, the 5^th^ and 95^th^ percentiles were then established for each of the measures. A Spearman rho non-parametric correlation analysis [37] was performed between ASO titers and each of the neuronal antibody titers and CaMKII values at each time point.

#### Subjects

Serial comparison: i) The Friedman non-parametric analysis for multiple measures per subject was used to detect differences between the longitudinal measurements [38]. ii) A calculation was then made per subject to determine whether their rise in titer or value had a four-fold elevation from the Pre-Exac 1 measurement compared to the four ensuing measurements. A four-fold or greater increase in antigen-specific antibody has been interpreted to indicate a significantly increased antibody production in response to vaccination and infection [39]. In this study, an example of a four-fold change is an increase in titer level of, e.g., 250 at Baseline (i.e. Pre-Exac 1) to 1000 at the exacerbation point.

Comparison to combined controls and to a published control group: i) Individual serial samples were evaluated to determine whether they exceeded the 95^th^ percentile identified in either the combined control group or a published control group modified by the removal of individuals with an ASO titer exceeding 400 units [3, 19]. The modified published control group was specifically included to provide a comparator for the multi-institution combined control group. ii) Samples were compared (Mann Whitney test, [40]) to determine whether ELISA titer values or CaMKII activation levels differed significantly from median values in either the combined controls or the published control groups titers/activity In the latter, case measures from each of the five time points (Pre-Exac 1 and 2, Exac, and Post Exac 1 and 2) were compared to the control groups (both combined and published) for each time point separately. iii) Lastly, Spearman rho correlations were evaluated between ASO and anti-DNase B values and the antibody titers (tubulin, lysoganglioside-GM1, D1 and D2 receptors) and CaMKII activation at each of the five time periods for the ExWS subjects. An adjustment of the p-values was made to provide an experiment-wise error rate of 0.05 across the six subject correlations with ASO and anti-DNase B at each time point.

## Results

### Controls

#### Comparison of Four Control Groups

Values for individual control groups 1–4 and the combined control population are shown in Table 4. Individual control values are presented in the S1 Table.

**Table 4:** Control Subjects: Four group and combined anti-neuronal antibody IgG titers.

	Group	N	Mean	Median	SD	Mean + 2 SD	Min	Max	5th %-tile	95^th^ %-tile
**Anti-Tubulin**	1	15	800	500	553	1,905	250	2,000	250	2,000
	2	17	647	500	235	1,117	500	1,000	500	1,000
	3	17	912	500	901	2,714	250	4,000	250	2,400
	4	21	1,762	1,000	1,044	3,850	1,000	4,000	1,000	4,000
	**Combined:**	**70**	**1,079**	**1,000**	**889**	**2,857**	**250**	**4,000**	**363**	**3,100**
**Anti-D2R**	1	15	6,667	4,000	5,273	17,214	2,000	16,000	2,000	16,000
	2	17	5,235	4,000	3,734	12,703	1,000	16,000	1,800	9,600
	3	17	9,912	8,000	10,823	31,557	500	32,000	1,700	32,000
	4	21	6,905	4,000	6,633	20,170	1,000	32,000	2,000	16,000
	**Combined:**	**70**	**7,179**	**4,000**	**7,186**	**21,550**	**500**	**32,000**	**2,000**	**24,800**
**Anti-D1R**	1	15	1,033	1,000	550	2,133	500	2,000	500	2,000
	2	17	2,824	2,000	2,186	7,196	1,000	8,000	1,000	8,000
	3	17	3,265	2,000	2,526	8,316	500	8,000	900	8,000
	4	21	4,810	2,000	5,066	14,941	1,000	16,000	1,000	16,000
	**Combined:**	**70**	**3,143**	**2,000**	**3,454**	**10,050**	**500**	**16,000**	**500**	**8,000**
**Anti-Lyso GM1**	1	14	119	90	66	251	80	320	80	216
	2	17	249	160	303	856	80	1,280	80	768
	3	17	424	320	298	1,019	80	1,280	80	768
	4	21	735	640	476	1,687	80	1,280	160	1,280
	**Combined:**	**69**	**414**	**320**	**408**	**1,230**	**80**	**1,280**	**80**	**1,280**
**CaMKII**	1	15	92	94	12	115	53	100	76	99
	2	12	89	88	12	112	72	112	76	108
	3†	—	—	—	—	—	—	—	—	—
	4	10	124	117	36	196	83	184	86	175
	**Combined:**	**37**	**100**	**94**	**26**	**151**	**53**	**184**	**78**	**157**
**ASO**	1	15	148	166	96	340	25	320	25	286
	2	15	96	63	82	260	25	250	25	250
	3	17	179	188	104	387	11	335	21	316
	4	16	84	42	94	271	25	370	25	223
	**Combined:**	**63**	**128**	**95**	**100**	**329**	**11**	**370**	**25**	**308**

Control sera were obtained from several different institutions: Group 1 were provided from the National Institute of Mental Health, Bethesda, MD and the Yale Child Study Center, New Haven CT. Group 2 were provided from the Yale Child Study Center; Group 3 from the Shaare Zedek Medical Center, Jerusalem, Israel; Group 4 from the Johns Hopkins Hospital.

Abbreviations: D2R (Dopamine 2 receptor), D1R (Dopamine 1 receptor), Lyso-GM1 (Lysoganglioside-GM1), CaMKII (Calcium calmodulin-dependent protein kinase II), ASO (antistreptolysinO).

† Insufficient sera available to perform this assay.

Anti-Tubulin: The Kruskal-Wallis test showed a significant difference in anti-tubulin antibodies among the four control groups (p<0.001). Subsequent multiple comparisons indicated that Group 4 had significantly higher values than the other three groups. The four-group combined 95^th^ percentile titer was 3,100 and median titer was 1,000. The published control group (Group 1) 95^th^ percentile titer was 2,000 and median titer was 500.

Anti-D2 receptor (D2R): No significant differences were identified among groups (p = 0.65). The four-group 95^th^ percentile titer was 24,800 and median titer was 4,000.

The published control group (Group 1) 95^th^ percentile titer was 16,000 and median titer was 4,000.

Anti-D1 receptor (D1R): Comparison of the four control groups showed a significant difference (p≤0.001) with Group 1 having significantly lower anti-D1R titers than the other groups. The four-group 95^th^ percentile titer was 8,000 and median titer was 2,000 for groups 2, 3 and 4. The published control group (Group 1) 95^th^ percentile titer was 2,000 and median titer was 1,000.

Anti-Lysoganglioside-GM1: Comparison of the four control groups showed a significant difference (p≤0.001) in anti-lysoganglioside-GM1 titers. Group 3 had significantly higher values than Group 1, and Group 4 had significantly higher values than Groups 1 and 2. The four-group 95^th^ percentile titer was 1280 and median titer was 320 (group 2 = 160, group 3 = 320, group 4 = 640). The published control group (Group 1) 95th percentile titer was 216 and median titer was 90.

CaMKII activation: Insufficient sera were available to perform this assay in control Group 3. Based on data from the three available control populations, the Kruskal-Wallis test indicated a significant difference between the groups (p = 0.021), with Group 4 having significantly higher values than Groups 1 and 2. The three-group 95^th^ percentile for activation was 157 and median CaMKII value was 94. The published control group (Group 1) 95^th^ percentile titer was 99 and median titer was 94.

ASO: Group comparisons indicated a significant difference (p = 0.017), with Group 3 having higher ASO values than Groups 2 and 4 suggesting more streptococcal infections in control Group 3. The four-group 95th percentile ASO titer was 308 and median titer was 95. The published control group (Group 1) 95th percentile ASO titer was 286 and median ASO titer was 166. No anti-DNase B values were available in controls.

### Longitudinal serum samples

#### Timing

**Fig 1:**
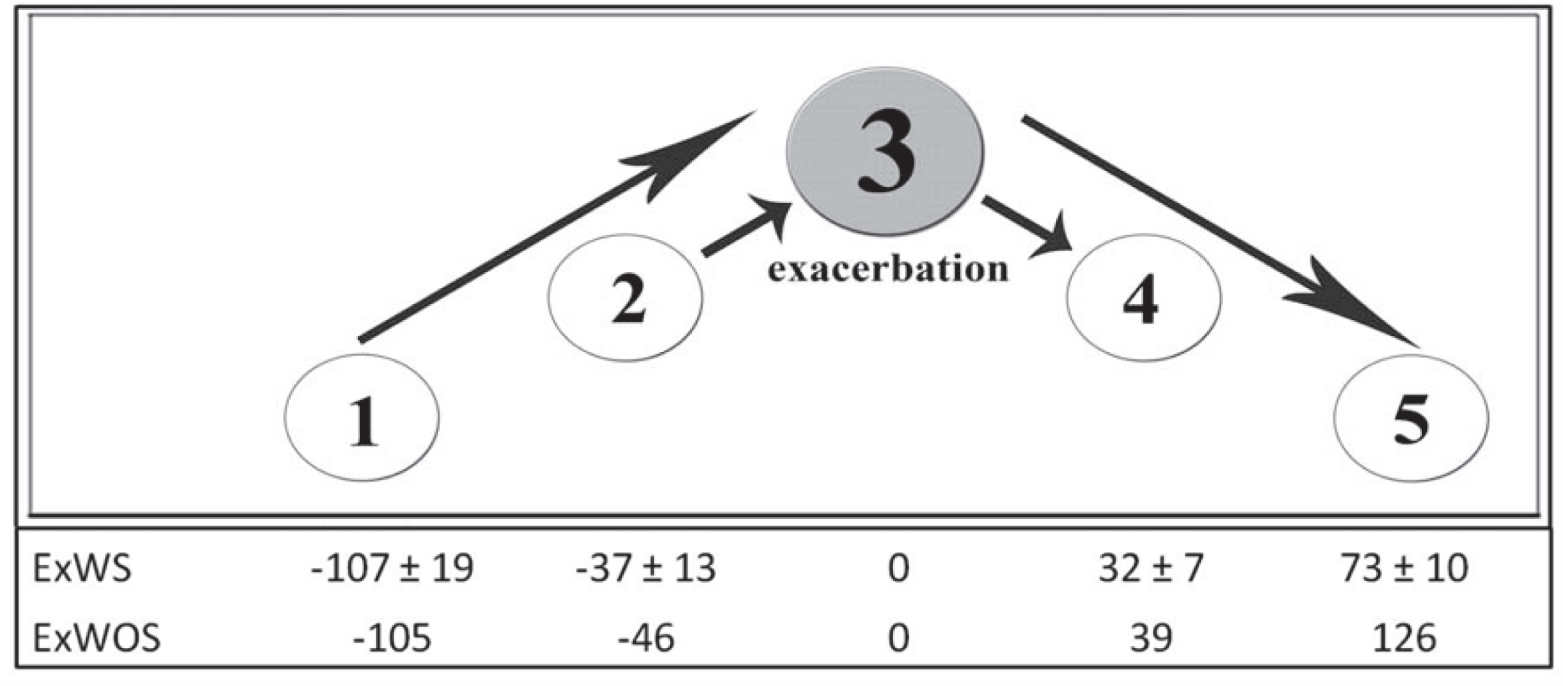
Diagram of sampling time points. Number of days (mean ± SD) from the exacerbation point for serum samples evaluated in the ExWS (exacerbation with streptococcal infection) and ExWOS (exacerbation without streptococcal infection) groups. Time points: 1 (Pre-Exac 1), 2 (Pre-Exac 2), 3 (Exac), 4 (Post-Exac 1), and 5 (Post-Exac 2). Adapted from [14].

The exacerbation sample (designated time ‘3’) was drawn within the period of clinical worsening and no more than two weeks from its onset. The mean timing of the Pre- and Post-Exac samples (mean ± SD) for the ExWS and ExWOS groups are shown in Fig. 1.

### Assay results for tic and/or OCD exacerbations associated with a streptococcal infection (ExWS)

**Fig 2:**
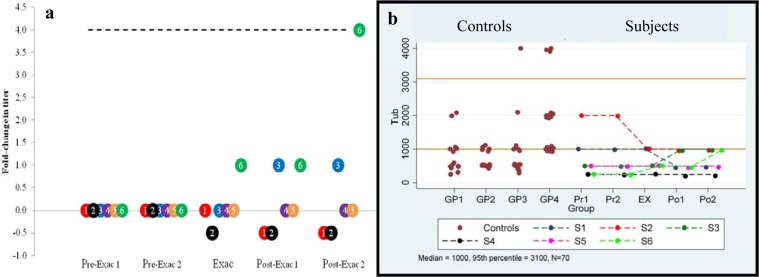
Anti-tubulin autoantibodies. a) Longitudinal anti-tubulin serum IgG titer fold-change from baseline in PANDAS-chronic tics and OCD subjects having a clinical exacerbation associated with a streptococcal infection. Baseline (Pre-Exac 1) for each individual subject is set at “0”. Subsequent points indicate changes in titer level. A four-fold-rise (----) is equivalent to increase in titer level, e.g., 250 to 1000. Subject number is presented within each circle. b) Longitudinal anti-tubulin serum titers compared to controls. Control values for Groups 1–4 are shown (**●**). The top and lower solid lines indicate the combined control group’s 95^th^ percentile and median, respectively. Actual serial values are presented for subjects 1–6 (1 = red, 2 = black, 3 = blue, 4 = purple, 5 = orange, and 6 = green).

Anti-Tubulin: Serial comparison between time points. Serial antibody titers are shown in Table 5 and Fig. 2A shows the fold-change in antibody IgG titer from baseline (i.e., Pre-Exac 1) to all of the ensuing time points for six ExWS subjects 1–6. Based on the Friedman test, there was no significant difference in the values between the five time points. No subject showed a four-fold increase in titers from baseline to the exacerbation point; although subject #6 increased four-fold between baseline and Post-Exac 2.

**Table 5:** Longitudinal anti-neuronal antibody IgG titers in PANDAS-chronic tics and OCD subjects with an exacerbation associated with a streptococcal infection (ExWS).

Subject	Antigen	Pre-Exac 1	Pre-Exac 2	Exac	Post-Exac 1	Post-Exac 2
1	Tubulin	1000	1000	1000	500	500
	D2R	4000	4000	4000	4000	4000
	D1R	4000●	4000●	4000●	2000●	2000●
	Lyso-GM1	160	160	160	640●	1280● ♦
2	Tubulin	2000●	2000●	1000	1000	1000
	D2R	4000	4000	4000	2000	4000
	D1R	4000●	4000●	8000● ♦	4000●	4000●
	Lyso-GM1	1280● ♦	1280● ♦	1280● ♦	1280● ♦	1280● ♦
3	Tubulin	500	500	500	1000	1000
	D2R	4000	4000	4000	4000	2000
	D1R	4000●	4000●	4000●	8000● ♦	8000● ♦
	Lyso-GM1	640●	640●	640●	640●	640●
4	Tubulin	250	250	250	250	250
	D2R	2000	2000	2000	2000	2000
	D1R	1000	1000	1000	1000	2000●
	Lyso-GM1	640●	320●	320●	320●	320●
5	Tubulin	500	500	500	500	500
	D2R	4000	8000	8000	8000	8000
	D1R	2000●	2000●	4000●	2000●	4000●
	Lyso-GM1	320●	640●	640●	640●	640●
6	Tubulin	250	250	500	500	1000
	D2R	2000	1000	2000	2000	2000
	D1R	1000	1000	1000	1000	2000●
	Lyso-GM1	640●	320●	640●	640●	320●

Abbreviations: Exac = exacerbation point; lyso-GM1 = lysoganglioside-GM1

♦ Titers equal to or exceeding the combined control group’s 95^th^ percentile antibody titers for tubulin (3100), D2R (24,800), D1R (8,000), and lysoganglioside-GM1 (1,280).

● Titers equal to or exceeding the published control group’s 95^th^ percentile antibody titers for tubulin (2000), D2R (16,500), D1R (2000), and lysoganglioside-GM1 (216).

Anti-Tubulin: Comparison of each time point to combined controls and a published control group. No individual had an antibody titer that was equal to or exceeded the 95th percentile titer of combined controls (3100; see Table 5 and Fig. 2B). Titers compared to a published control groups’ 95^th^ percentile (Group 1: 2,000), were elevated at least once in one subject. The anti-tubulin antibody titers of the six children at each of the five time points (Pre-Exac 1 and 2, Exac, and Post Exac 1 and 2) were not significantly different from the combined controls’ median values (titer of 1000) (p = 0.22/0.22/0.2/0.2/0.41, respectively) or the published control group’s median values (Group 1 median titer of 500) (p = 0.52/0.52/0.99/0.99/0.99, respectively) at each time point.

**Fig 3:**
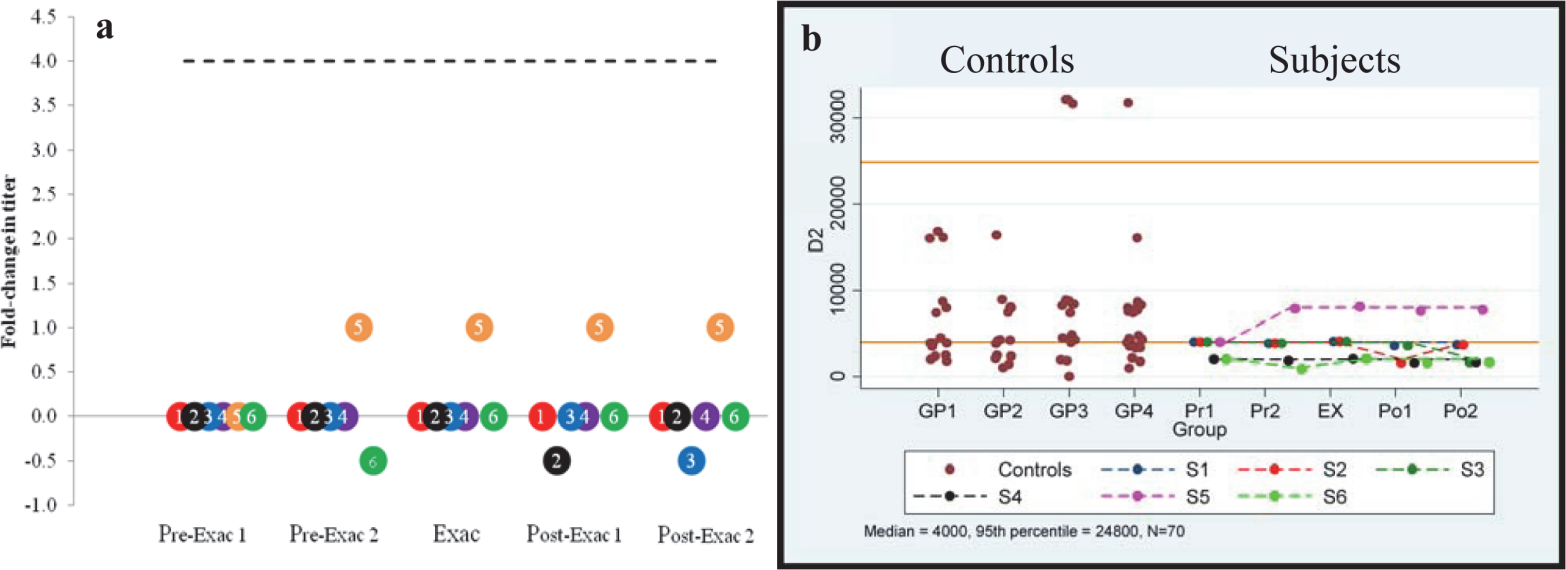
Anti-dopamine D2 autoantibodies. a) Longitudinal anti-dopamine D2 receptor serum IgG titer fold-change from baseline in PANDAS-chronic tics and OCD subjects having a clinical exacerbation associated with a streptococcal infection. Baseline (Pre-Exac 1) for each individual subject is set at “0”. Subsequent points indicate changes in titer level. A four-fold-rise (----) is equivalent to increase in titer level, e.g., 250 to 1000. Subject number is presented within each circle. b) Longitudinal anti-dopamine D2 receptor serum titers compared to controls. Control values for Groups 1–4 are shown (**●**).The top and lower solid lines indicate the combined control group’s 95^th^ percentile and median, respectively. Actual serial values are presented for subjects 1–6. (1 = red, 2 = black, 3 = blue, 4 = purple, 5 = orange, and 6 = green).

Anti-Dopamine D2 receptor (D2R): Serial comparison between time points. Serial values are shown in Table 5 and Fig. 3A shows the fold-change in titer from baseline (Pre-Exac 1) to all of the ensuing points (Pre-Exac 2, Exac, Post-Exac 1, and Post-Exac 2) for ExWS subjects 1–6. Based on the Friedman test, there was no significant difference in the values between the five time points. No subject had a four-fold increase between baseline and the exacerbation point.

Anti-Dopamine D2 receptor (D2R): Comparison of each time point to combined controls and a published control. No individual’s antibody titer was equal to or exceeded the 95th percentile titer of the combined control group (24,800, Table 5 and Fig. 3B) or the published control group’s 95^th^ percentile (Group 1; 16,000). The anti-D2R antibody titers at any of the five time points (Pre-Exac 1 and 2, Exac, and Post Exac 1 and 2) were not significantly different from the combined controls’ median values (median titer of 4,000) or the published control group’s median values (Group 1 median titer of 4,000).

**Fig 4:**
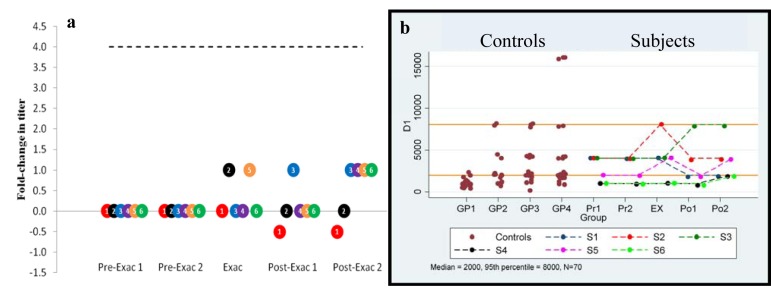
Anti-dopamine D1 autoantibodies. a) Longitudinal anti-dopamine D1 receptor serum IgG titer fold-change from baseline in PANDAS-chronic tics and OCD subjects having a clinical exacerbation associated with a streptococcal infection. Baseline (pre-Exac 1) for each individual subject is set at “0”. Subsequent points indicate changes in titer level. A four-fold-rise (----) is equivalent to increase in titer level, e.g., 250 to 1000. Subject number is presented within each circle. b) Longitudinal anti-dopamine D1 receptor serum titers compared to controls. Control values for Groups 1–4 are shown (**●**).The top and lower solid lines indicate the combined control group’s 95^th^ percentile and median, respectively. Actual serial values are presented for subjects 1–6. (1 = red, 2 = black, 3 = blue, 4 = purple, 5 = orange, and 6 = green).

Anti-Dopamine D1 receptor (D1R): Serial comparison between time points. Serial values are shown in Table 5 and Fig. 4A shows fold-change in titer from baseline to each of the ensuing points. Based on the Friedman test, there was no significant difference in the values between the five time points. No subject had a four-fold increase between baseline and the exacerbation point.

Anti-Dopamine D1 receptor (D1R): Comparison of each time point to combined controls and a published control group. An individual antibody titer was equal to or exceeded the 95th percentile titer of the combined control group (8,000) in two subjects #2 and #3 (three samples) (Table 5 and Fig. 4B) and exceeded the published control group’s 95^th^ percentile (Group 1: 2,000) in all samples from subjects 1, 2, 3, and 5 and the Post-Exac 2 point in subjects 4 and 6. Comparison of the anti-D1R titers of the six children at the five time points (Pre-Exac 1 and 2, Exac, and Post Exac 1 and 2) were not significantly different from the combined control group’s median values (median of 2,000), but were significantly different (p<0.01) at all points compared to the published control group’s median values (Group 1 median titer of 1,000).

**Fig 5:**
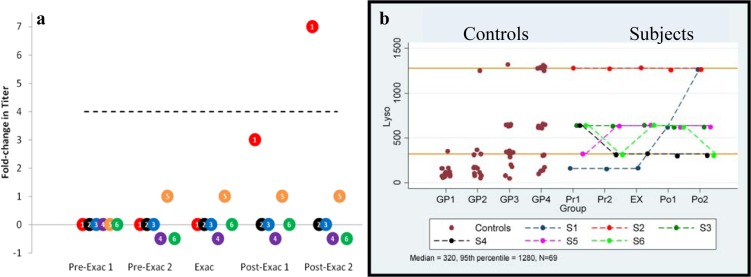
Anti- lysoganglioside-GM1 autoantibodies. a) Longitudinal anti-lysoganglioside-GM1 serum IgG titer fold-change from baseline in PANDAS-chronic tics and OCD subjects having a clinical exacerbation associated with a streptococcal infection. Baseline (pre-Exac 1) for each individual subject is set at “0”. Subsequent points indicate changes in titer level. A four-fold-rise (----) is equivalent to increase in titer level, e.g., 250 to 1000. Subject number is presented within each circle. b) Longitudinal anti- lysoganglioside-GM1 receptor serum titers compared to controls. Control values for Groups 1–4 are shown (**●**).The top and lower solid lines indicate the combined control group’s 95^th^ percentile and median, respectively. Actual serial values are presented for subjects 1–6. (1 = red, 2 = black, 3 = blue, 4 = purple, 5 = orange, and 6 = green).

Anti-Lysoganglioside-GM1: Serial comparison between time points. Serial values are shown in Table 5 and Fig. 5A shows fold-change in titer from baseline to each of the ensuing points. Based on the Friedman test, there was no significant difference in the values between the five time points. No subject had a four-fold increase between baseline and the exacerbation point. Only one subject (#1) had a greater than four-fold increase in titer, from the exacerbation point progressing to Post-Exac 2.

Anti-Lysoganglioside-GM1: Comparison of each time point to combined controls and a published control group. An individual’s antibody titer was equal to or exceeded the 95th percentile titer of combined control group’s titer of 1280 in two subjects (six samples); includes five time points in a single subject (Table 5 and Fig. 5B). When compared to the published control group’s 95^th^ percentile (Group 1: 216), values were higher in all samples from subjects 2–6, and the Post-Exac 1 and 2 points in subject 1. Comparison of the anti-lysoganglioside-GM1 antibody titers of the six children at the five time points vs the combined control group’s median values (median titer of 320) showed that they were significantly different at the Post Exac 1 and 2 time points (p = 0.08/0.12/0.08/0.02/0.03, respectively) and the other 3 time points approached significance. Compared to the published control group’s median values (Group1 median titer of 90), all time points were significantly different (p<0.001).

CaMKII activation *in ExWS and ExWOS*: Serial comparison between time points. Serial values for the CaMKII activity in individual PANDAS patients having an exacerbation correlating with a streptococcal infection (ExWS, subjects 1–6) and an exacerbation without a streptococcal infection (ExWOS, subjects 7 and 8) are shown in Table 6. Insufficient sera resulted in at least one missing time-point in five of the eight subjects. Based on the Friedman test, there was no significant difference in the values between the five time points.

**Table 6:** Longitudinal CaMKII antibody mediated neuronal cell signaling in sera from PANDAS-chronic tics and OCD subjects (percentage above baseline).

**Subjects with an exacerbation associated with a streptococcal infection (ExWS)**						
	**Subject**	**Pre-Exac 1**	**Pre-Exac 2**	**Exac**	**Post-Exac 1**	**Post-Exac 2**
CaMKII	1	120●	n/a	89	104●	175● ♦
	2	161● ♦	n/a	195● ♦	155●	156●
	3	153●	130●	168● ♦	n/a	n/a
	4	157● ♦	161● ♦	167● ♦	180● ♦	171● ♦
	5	n/a	n/a	205● ♦	n/a	203● ♦
	6	176● ♦	173●	162● ♦	171● ♦	130●
	Mean + SD	153 ± 21	145 ± 16	164 ± 41	153 ± 34	167 ± 27
**Subjects with an exacerbation without an associated streptococcal infection (ExWOS)**						
	Subject	Pre-Exac 1	Pre-Exac 2	Exac	Post-Exac 1	Post-Exac 2
CaMKII	7	125●	n/a	133●	170● ♦	122●
	8	139●	135●	109●	104●	113●
	Mean	132	135	121	137	117

♦ Indicates a value equal to or exceeding the combined control’s 95^th^ percentile (157).

● Indicates a value equal to or exceeding the published control group 95^th^ percentile (99).

n/a = not available.

**Fig 6:**
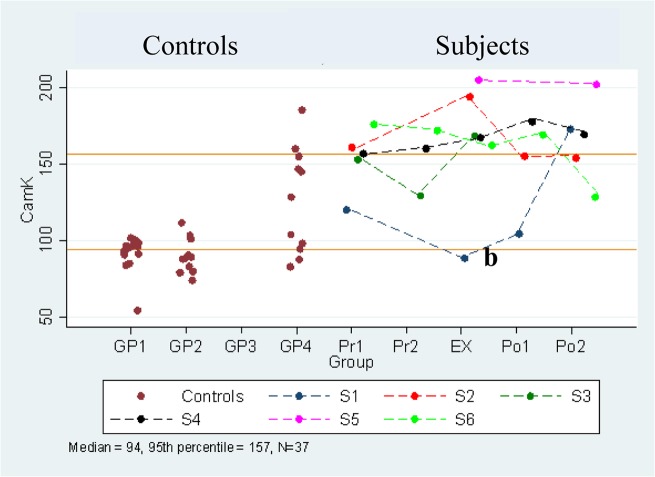
Longitudinal CAMKII activity. Longitudinal CAMKII activity in PANDAS-chronic tics and OCD compared to controls. Control values for Groups 1–4 are shown (**●**). The top and lower solid lines indicate the combined control group’s 95^th^ percentile and median, respectively. Actual serial values are presented for 7 subjects (1 = red, 2 = black, 3 = blue, 4 = purple, 6 = green, 7 = olive green, and 8 = dark red).

CaMKII activation in ExWS and ExWOS: Comparison of each time point to combined controls and a published control group. For antibody-induced CaMKII activity (8 subjects, 6 ExWS and 2 ExWOS), an individual value was equal to or exceeded the combined control group’s (n = 37) 95th percentile (157) in seven subjects (total of 13 samples). Five subjects had increased values at the time of clinical exacerbation (see asterisks in Table 6 and Fig. 6). When compared to the published control groups’ 95^th^ percentile (Group 1; 99), values were higher in all available samples from all subjects, except for subject 1 at their Exac point. The CaMKII activation values of the serum of the eight children at the five time points (Pre-Exac 1 and 2, Exac, and Post Exac 1 and 2) vs the combined controls’ median values (median 94) as well as the published control group’s median values (Group 1, median 94) were significantly different at all-time points (p<0.002), respectively.

### Correlations of antibody titers/activation activity with ASO and anti-DNase B levels

ASO and anti-DNase B levels of the test subjects are shown in S2 Table. Spearman correlation analysis across the ExWS subjects showed that at any of the five time periods, there was no statistically significant association between the ASO titer and the other titers from anti-neuronal antibody assays or neuronal cell signaling assay. Anti-DNase B levels also showed no correlation with anti-neuronal titers or CaMKII activation.

## Discussion

Serial sample evaluations provide the essential opportunity for comparison of clinical exacerbations and antibody levels [32]. In a comparison between time points, neither the Friedman test nor changes in titer fold increases, compared to the Pre-Exac 1 value, showed an association between clinical exacerbation and rises in antibody reactivity against tubulin, lysoganglioside-GM1, D1 and D2 receptors or changes in CaMKII activity. Although a four-fold change in titer between pre-exacerbation and exacerbation points has been used as a measure of change, it is unclear whether a higher or lower alteration provides the optimal degree of significance. These results are similar to prior longitudinal studies in this population evaluating antibodies against human striatal and frontal lobe brain tissue, inflammatory cytokines, and ELISA optical density measurements of antibodies against tubulin and the D2 receptor [31].

A second aim was to compare individual subject serum antibody titer/activity levels to controls. This comparison has clinical relevance, recognizing that elevated antibodies can cross the blood brain barrier and target neurons *in vivo* [3] and an infectious process can enhance antibody passage into the brain [41]. The number of controls evaluated in this protocol (n = 70), exceeds that in prior published autoantibody studies measuring similar biomarkers in SC and PANDAS-choreiform (Tables 1 and 2). Significant differences for tubulin, D1R, and lysoganglioside-GM1 titers, and CaMKII activity were identified among the four institutional control groups. Control groups 1 and 2 tended to have the lowest values, although whether this is related to fewer streptococcal infections [2, 3, 16, 19] or associated familial disorders is unclear. Hence, after establishing a formal exclusionary ASO titer of 400, all comparisons with study subjects were made against both the titer/activity level from the combined control group and the value from a previously published control (Group 1) [2, 3, 16, 19]. Future studies should be performed in precisely defined controls in different locations, in order to determine whether there is a meaningful regional variability of anti-neuronal antibodies.

Although antibody-induced CaMKII activation did not correlate with clinical exacerbations, individual serum values were increased in the midst of exacerbation in 5/6 ExWS subjects and median levels were significantly elevated from controls at all-time points. Elevated CaMKII activation has also been identified in SC patients [2, 17, 22] and in PANDAS-choreiform [22]. Since the assay utilizes serum, a potential limitation is the possibility that serum containing drugs or other factors could affect the results. CaMKII, one of two subclasses of calcium-calmodulin kinases, is highly expressed in the striatum [42] and its role in movement and neuropsychiatric disorders could involve either postsynaptic or presynaptic activity [43–45]. Further studies are required to clarify the pathophysiological role of CaMKII activity and to determine whether it is a biological marker in SC and PANDAS-choreiform as well as in our new group with chronic tics and OCD.

In this study, results show that children with PANDAS-chronic tics and OCD do not have abnormally elevated antibody titers against D2R and tubulin. These findings are supported by single-point-in-time and longitudinal measurements in a cohort of similar subjects previously investigated with ELISA optical density testing [31]. Outcomes are, however, different from elevated ELISA titers found in SC [3, 16, 19] and PANDAS-choreiform cases [3, 19] (Tables 1 and 2). Antibody-mediated signaling of D2R on human D2R transfected cell lines has been reported for sera from SC and PANDAS-choreiform patients [3], but was not evaluated in this study. Anti-tubulin antibodies are not increased in PANDAS-chronic tics and OCD, based on the current and a prior report [31], but are in some [17, 18], but not all [16] children with SC.

Outcomes from anti-lysoganglioside-GM1 and D1R studies vary depending on the control group (combined or published) and statistical comparison (95^th^ percentile or median level at each time point) used for comparison. For example, in PANDAS-chronic tics and OCD subjects anti-lysoganglioside-GM1 titers were elevated in 6/30 samples (five times in subject #2) when compared to the combined controls’ 95^th^ percentile, but in 27/30 samples when compared to the published control group (Group 1). Further, when time point values were contrasted to combined control median levels they were statistically different at Post-Exac 1 and 2, but differed at all-time points compared to the published control (Group 1). Similar discrepancies were also seen for anti-D1R antibody titers: only 3/30 individual samples were at or above the combined control group’s 95^th^ percentile, whereas 22/30 were at or above the published control group’s (Group 1) 95^th^ percentile; and comparisons to median levels showed differences only when compared to published controls. Variable interpretations of anti-lysoganglioside-GM1 data, based on the selection of controls has also been reported in SC [16, 17]. In a study of PANDAS-chronic tics and OCD, 7/12 had a positive competitive inhibition ELISA assay with lysoganglioside GM1, but only one showed an alteration in binding that directly correlated with a clinical exacerbation [14].

Results of autoantibody testing raise the possibility of at least two differing subgroups of children fulfilling the original proposed criteria for PANDAS [5]. One group, displaying choreiform piano-playing movements (PANDAS-choreiform), appears to be very similar to SC: both have movements in the chorea spectrum [9], elevated ELISA titer measurements of anti-D1R and anti-D2R IgG [3, 16, 19], increased CaMKII activity [2, 17, 22], and possibly elevated anti-lysoganglioside-GM1 [17, 22]. A second potential group (PANDAS-chronic tics and OCD) includes children with at least two prior acute fulminant episodes of tics or OCD in a temporal association with a GABHS infection, but having absent or unmentioned piano-playing movements. Children with PANDAS-chronic tics and OCD show increased CaMKII activation, normal levels of antibodies to tubulin and D2R, and control group-dependent alterations of anti-D1R and lysoganglioside-GM1.

Attempts to confirm the presence of anti-neuronal antibodies in the sera of children with SC and PANDAS (Tables 1 and 2) have included both ELISA assays (IgG binding to commercial antigens) and flow-cytometry-based detection; the latter involving antibody binding to live cells expressing the candidate antigens in their membrane-bound conformation [3, 21, 23]. Results, however, are not always equivalent. For example: a) ELISA methodology has detected anti-D2R antibodies in sera from PANDAS-choreiform and SC patients; b) antibodies from both study groups signaled D2 dopamine receptors in a transfected human fibroblast L cell line [3]; but c) D2 receptors transfected in HEK293 cells were bound by antibodies in either some [21] or all [3] children with SC and none with PANDAS [3, 21]. Variability is also reported with transfected D1 receptors: absent binding in both SC and PANDAS to HEK293 cells [21], but binding activity is identified in human fibroblast L cells expressing the D1 receptor in PANDAS-choreiform subjects (Zuccolo and Cunningham, personal communication). Lastly, immunoglobulin (IgG) surface binding to differentiated SH-SY5Y cells (cells with neuronal and dopaminergic characteristics) were not increased in PANDAS [23]. Future studies are clearly required to determine the epitopes, the basis for the different results, and to better understand the role and mechanisms of antibodies in disease conditions.

This study joins other reports in raising questions about the association of GABHS in cases with recurrent, dramatic exacerbations of tics and OCD, but lacking the fine piano-playing choreiform movements [10, 11, 46–49]. In our subjects, ASO and anti-DNase B showed no positive correlation with anti-neuronal antibody titers or antibody-mediated CaMKII activity at each of the five time points. These data differ from that in SC where an association has been identified between ASO titers and antibodies against the dopamine D1 and D2 receptor [16]. Animal models have also been used to support an association between streptococcal antibodies and disease symptoms [19, 20, 50]. To our knowledge, there has never been a study attempting to correlate streptococcal antibodies and anti-neuronal antibody titers or CaMKII activity in the PANDAS-choreiform cohort. This study does not address any potential association between GABHS infection and autoimmunity as an explanation for the initial onset of symptoms; all participating subjects having had at least two dramatic symptom exacerbations.

In summary, biomarker differences were identified in the anti-neuronal antibody profiles in PANDAS-chronic tics and OCD from that reported in PANDAS-choreiform, the latter being similar to Sydenham chorea. Limitations in this study include the small number of subjects with streptococcal associated clinical exacerbations, an insufficient volume of sera to perform CaMKII assays at all-time points in subjects and in all controls, insufficient knowledge regarding the timing of maximum immune responses and antibody kinetics, and the lack of flow-cytometry-based detection of antibody binding. Even noting these deficits, the analysis of a larger more diverse group of controls and serial prospective PANDAS-chronic tics and OCD samples provides important information in the study of possible autoimmune neurological disorders. Our identification of variable titers in serial blood samples emphasizes the limitation of single-point-in-time analyses, highlights the need for correlating sample timing with the clinical exacerbation, and enhances the requirement for longitudinal analyses. Data confirm that the interpretation of individual antibody titers or CaMKII induced activity is influenced by the control group selected for comparison. Requirements for all controls should include comprehensive clinical evaluations and standardized laboratory assessments for concurrent infections.

Several clinical investigators have advocated for a reassessment of all childhood acute neuropsychiatric disorders based on a separation into monophasic and recurrent symptom categories, downgrading the presence of tics, eliminating a specific association with a particular organism, and requiring a comprehensive etiological investigation [13, 15]. If an immunological role in any disorder is to be identified, it is essential that serial serum samples be obtained in adequate numbers of well-characterized patients and appropriate controls.

## Supporting Information

S1 Table Individual control data. (PDF)

S2 Table Anti-streptococcal ASO and anti-DNase B antibodies in longitudinal PANDAS-tics and OCD subjects. (PDF)

## References

[1] Galvin JE, Hemric ME, Ward K, Cunningham MW. Cytotoxic mAb from rheumatic carditis recognizes heart valves and laminin. J Clin Invest. 2000;106: 217–224. 10903337 10.1172/JCI7132 PMC314302

[2] Kirvan CA, Swedo SE, Kurahara D, Cunningham MW. Streptococcal mimicry and antibody-mediated cell signaling in the pathogenesis of Sydenham's chorea. Autoimmunity. 2006a;39: 21–29. 16455579 10.1080/08916930500484757

[3] Cox CJ, Sharma M, Leckman JF, Zuccolo J, Zuccolo A, Kovoor A, et al Brain Human Monoclonal Autoantibody from Sydenham Chorea Targets Dopaminergic Neurons in Transgenic Mice and Signals Dopamine D2 Receptor: Implications in Human Disease. The Journal of Immunology. 2013;191: 5524–5541. 10.4049/jimmunol.1102592 24184556 PMC3848617

[4] Cardoso F. Sydenham's chorea Handb Clin Neurol. Elsevier B.V.: Amsterdam, The Netherlands; 2011 pp. 221–229. 10.1016/B978-0-444-52014-2.00014-8 21496581

[5] Swedo SE, Leonard HL, Garvey M, Mittleman B, Allen AJ, Perlmutter S, et al Pediatric autoimmune neuropsychiatric disorders associated with streptococcal infections: clinical description of the first 50 cases. Am J Psychiatry. 1998;155: 264–271. 9464208 10.1176/ajp.155.2.264

[6] Kurlan R. Tourette's syndrome and 'PANDAS': will the relation bear out? Pediatric autoimmune neuropsychiatric disorders associated with streptococcal infection. Neurology. 1998;50: 1530–1534. 9633690 10.1212/wnl.50.6.1530

[7] Singer HS, Loiselle C. PANDAS: a commentary. J Psychosom Res. 2003;55: 31–39. 12842229 10.1016/s0022-3999(02)00582-2

[8] Kurlan R. The PANDAS hypothesis: losing its bite? Mov Disord. 2004;19: 371–374. 15077234 10.1002/mds.20107

[9] Kurlan R, Kaplan EL. The pediatric autoimmune neuropsychiatric disorders associated with streptococcal infection (PANDAS) etiology for tics and obsessive-compulsive symptoms: hypothesis or entity? Practical considerations for the clinician. Pediatrics. 2004;113: 883–886. 15060240 10.1542/peds.113.4.883

[10] Kurlan R, Johnson D, Kaplan EL, Group TSS. Streptococcal infection and exacerbations of childhood tics and obsessive-compulsive symptoms: a prospective blinded cohort study. Pediatrics. 2008;121: 1188–1197. 10.1542/peds.2007-2657 18519489

[11] Leckman J, King RA, Gilbert DL, Coffey BJ, Singer HS, Dure LSt, et al Streptococcal upper respiratory tract infections and exacerbations of tic and obsessive-compulsive symptoms: A prospective longitudinal study. J Am Acad Child Adolesc Psychiatry. 2011;50: 108–118. 10.1016/j.jaac.2010.10.011 21241948 PMC3024577

[12] Singer HS. Tourette syndrome and other tic disorders Handbook of Clinical Neurology. Amsterdam, The Netherlands: Elsevier B.V.; 2011 pp. 641–657. 10.1016/B978-0-444-52014-2.00046-X 21496613

[13] Singer HS, Gilbert DL, Wolf DS, Mink JW, Kurlan R. Moving from PANDAS to CANS. J Pediatr. 2012;160: 725–731. 10.1016/j.jpeds.2011.11.040 22197466

[14] Singer HS, Gause C, Morris C, Lopez P. Serial immune markers do not correlate with clinical exacerbations in pediatric autoimmune neuropsychiatric disorders associated with streptococcal infections. Pediatrics. 2008;121: 1198–1205. 10.1542/peds.2007-2658 18519490

[15] Swedo SE, Leckman J, Rose NR. From Research Subgroup to Clinical Syndrome: Modifying the PANDAS Criteria to Describe PANS (Pediatric Acute-onset Neuropsychiatric Syndrome). Pediatr Therapeut. 2012;2: 113–122.

[16] Ben-Pazi H, Stoner JA, Cunningham MW. Dopamine Receptor Autoantibodies Correlate with Symptoms in Sydenham's Chorea. PloS one. 2013;8: e73516 10.1371/journal.pone.0073516 24073196 PMC3779221

[17] Kirvan CA, Swedo SE, Heuser JS, Cunningham MW. Mimicry and autoantibody-mediated neuronal cell signaling in Sydenham chorea. Nat Med. 2003;9: 914–920. 12819778 10.1038/nm892

[18] Kirvan CA, Cox CJ, Swedo SE, Cunningham MW. Tubulin is a neuronal target of autoantibodies in Sydenham's chorea. J Immunol. 2007;178: 7412–7421. 17513792 10.4049/jimmunol.178.11.7412

[19] Brimberg L, Benhar I, Mascaro-Blanco A, Alvarez K, Lotan D, Winter C, et al Behavioral, pharmacological, and immunological abnormalities after streptococcal exposure: a novel rat model of Sydenham chorea and related neuropsychiatric disorders. Neuropsychopharmacology. 2012;37: 2076–2087. 10.1038/npp.2012.56 22534626 PMC3398718

[20] Yaddanapudi K, Hornig M, Serge R, De Miranda J, Baghban A, Villar G, et al Passive transfer of streptococcus-induced antibodies reproduces behavioral disturbances in a mouse model of pediatric autoimmune neuropsychiatric disorders associated with streptococcal infection. Mol Psychiatry. 2010;15: 712–726. 10.1038/mp.2009.77 19668249

[21] Dale RC, Merheb V, Pillai S, Wang D, Cantrill L, Murphy TK, et al Antibodies to surface dopamine-2 receptor in autoimmune movement and psychiatric disorders. Brain. 2012;135: 3453–3468. 10.1093/brain/aws256 23065479

[22] Kirvan CA, Swedo SE, Snider LA, Cunningham MW. Antibody-mediated neuronal cell signaling in behavior and movement disorders. J Neuroimmunol. 2006b;179: 173–179. 16875742 10.1016/j.jneuroim.2006.06.017

[23] Brilot F, Merheb V, Ding A, Murphy T, Dale RC. Antibody binding to neuronal surface in Sydenham chorea, but not in PANDAS or Tourette syndrome. Neurology. 2011;76: 1508–1513. 10.1212/WNL.0b013e3182181090 21411742 PMC3087465

[24] Martino D, Dale RC, Gilbert DL, Giovannoni G, Leckman JF. Immunopathogenic mechanisms in tourette syndrome: A critical review. Movement Disorders. 2009;24: 1267–1279. 10.1002/mds.22504 19353683 PMC3972005

[25] Gause C, Morris C, Vernekar S, Pardo-Villamizar C, Grados MA, Singer HS. Antineuronal antibodies in OCD: comparisons in children with OCD-only, OCD+ chronic tics and OCD+ PANDAS. Journal of Neuroimmunology. 2009;214: 118–124. 10.1016/j.jneuroim.2009.06.015 19628285

[26] Dale RC, Candler PM, Church AJ, Wait R, Pocock JM, Giovannoni G. Neuronal surface glycolytic enzymes are autoantigen targets in post-streptococcal autoimmune CNS disease. J Neuroimmunol. 2005;172: 187–197. 16356555 10.1016/j.jneuroim.2005.10.014

[27] Church AJ, Dale RC, Lees AJ, Giovannoni G, Robertson MM. Tourette's syndrome: a cross sectional study to examine the PANDAS hypothesis. J Neurol Neurosurg Psychiatry. 2003;74: 602–607. 12700302 10.1136/jnnp.74.5.602 PMC1738462

[28] Singer HS, Hong JJ, Yoon DY, Williams PN. Serum autoantibodies do not differentiate PANDAS and Tourette syndrome from controls. Neurology. 2005;65: 1701–1707. 16207842 10.1212/01.wnl.0000183223.69946.f1

[29] Pavone P, Bianchini R, Parano E, Incorpora G, Rizzo R, Mazzone L, et al Anti-brain antibodies in PANDAS versus uncomplicated streptococcal infection. Pediatr Neurol. 2004;30: 107–110. 14984902 10.1016/S0887-8994(03)00413-2

[30] Morris CM, Pardo-Villamizar C, Gause CD, Singer HS. Serum autoantibodies measured by immunofluorescence confirm a failure to differentiate PANDAS and Tourette syndrome from controls. J Neurol Sci. 2009;276: 45–48. 10.1016/j.jns.2008.08.032 18823914

[31] Morris-Berry C, Pollard M, Gao S, Thompson C, Singer H. Anti-streptococcal, tubulin, and dopamine receptor 2 antibodies in children with PANDAS and Tourette syndrome: Single-point and longitudinal assessments. Journal of Neuroimmunology. 2013;264: 106–113. 10.1016/j.jneuroim.2013.09.010 24080310

[32] Johnson DR, Kurlan R, Leckman J, Kaplan EL. The human immune response to streptococcal extracellular antigens: clinical, diagnostic, and potential pathogenetic implications. Clin Infect Dis. 2010;50: 481–490. 10.1086/650167 20067422

[33] Kaplan EL, Rothermel CD, Johnson DR. Antistreptolysin O and anti-deoxyribonuclease B titers: normal values for children ages 2 to 12 in the United States. Pediatrics. 1998;101: 86–88. 9417157 10.1542/peds.101.1.86

[34] Shet A, Kaplan EL. Clinical use and interpretation of group A streptococcal antibody tests: a practical approach for the pediatrician or primary care physician. Pediatr Infect Dis J. 2002;21: 420–426. 12150180 10.1097/00006454-200205000-00014

[35] Johnson DR, Kaplan EL, Sramek J, Bicova R, Havlicek J, Havlickova H, et al Laboratory Diagnosis of Group A Streptococcal Infections. Geneva, Switzerland: World Health Organization; 1996. pp.

[36] Kruskal W, Wallis W. Use of Ranks in One-Criterion Variance Analysis. Journal of the American Statistical Association. 1952;47: 583–621.

[37] Spearman C. Demonstration of Formulæ for True Measurement of Correlation. The American Journal of Psychology. 1907;18: 161–169.

[38] Friedman M. The use of ranks to avoid the assumption of normality implicit in the analysis of variance. Journal of the American Statistical Association (American Statistical Association). 1937;32: 675–701.

[39] Zaccaro DJ, Wagener DK, Whisnant CC, Staats HF. Evaluation of vaccine-induced antibody responses: impact of new technologies. Vaccine. 2013;31: 2756–2761. 10.1016/j.vaccine.2013.03.065 23583812 PMC3672347

[40] Mann HB, Whitney DR. On a test of whether one of two random variables is stochastically larger than the other. The Annals of Mathematical Statistics. 1947;18: 50–60.

[41] Huerta PT, Kowal C, DeGiorgio LA, Volpe BT, Diamond B. Immunity and behavior: antibodies alter emotion. Proc Natl Acad Sci U S A. 2006;103: 678–683. 16407105 10.1073/pnas.0510055103 PMC1334673

[42] Erondu NE, Kennedy MB. Regional distribution of type II Ca2+/calmodulin-dependent protein kinase in rat brain. J Neurosci. 1985;5: 3270–3277. 4078628 10.1523/JNEUROSCI.05-12-03270.1985 PMC6565219

[43] Klug JR, Mathur BN, Kash TL, Wang HD, Matthews RT, Robison AJ, et al Genetic inhibition of CaMKII in dorsal striatal medium spiny neurons reduces functional excitatory synapses and enhances intrinsic excitability. PloS one. 2012;7: e45323 10.1371/journal.pone.0045323 23028932 PMC3448631

[44] Waxham MN, Malenka RC, Kelly PT, Mauk MD. Calcium/calmodulin-dependent protein kinase II regulates hippocampal synaptic transmission. Brain Res. 1993;609: 1–8. 8389645 10.1016/0006-8993(93)90847-g

[45] Padmanabhan S, Lambert NA, Prasad BM. Activity‐dependent regulation of the dopamine transporter is mediated by Ca2+/calmodulin‐dependent protein kinase signaling. European Journal of Neuroscience. 2008;28: 2017–2027. 10.1111/j.1460-9568.2008.06496.x 19046383

[46] Lin H, Williams KA, Katsovich L, Findley DB, Grantz H, Lombroso PJ, et al Streptococcal upper respiratory tract infections and psychosocial stress predict future tic and obsessive-compulsive symptom severity in children and adolescents with Tourette syndrome and obsessive-compulsive disorder. Biol Psychiatry. 2010;67: 684–691. 10.1016/j.biopsych.2009.08.020 19833320 PMC2843763

[47] Luo F, Leckman JF, Katsovich L, Findley D, Grantz H, Tucker DM, et al Prospective longitudinal study of children with tic disorders and/or obsessive-compulsive disorder: relationship of symptom exacerbations to newly acquired streptococcal infections. Pediatrics. 2004;113: e578–585. 15173540 10.1542/peds.113.6.e578

[48] Macerollo A, Martino D. Pediatric Autoimmune Neuropsychiatric Disorders Associated with Streptococcal Infections (PANDAS): An Evolving Concept. Tremor and other hyperkinetic movements (New York, NY). 2013;3: pii: tre-03-167-4158-4157. 10.7916/D8ZC81M1 PMC3783973 24106651

[49] Mohammad SS, Ramanathan S, Brilot F, Dale RC. Autoantibody-associated movement disorders. Neuropediatrics. 2013;44: 336–345. 10.1055/s-0033-1358603 24203856

[50] Lotan D, Benhar I, Alvarez K, Mascaro-Blanco A, Brimberg L, Frenkel D, et al Behavioral and neural effects of intra-striatal infusion of anti-streptococcal antibodies in rats. Brain, Behavior, and Immunity. 2014;38: 249–262. 10.1016/j.bbi.2014.02.009 24561489 PMC4000697

