# Supplementary material for: Neuronal Antibody Biomarkers for Sydenham’s Chorea Identify a New Group of Children with Chronic Recurrent Episodic Acute Exacerbations of Tic and Obsessive Compulsive Symptoms Following a Streptococcal Infection
Source: PLoS One. 2015 Mar 20;10(3):e0120499. doi: 10.1371/journal.pone.0120499 (PMC4368605; doi:10.1371/journal.pone.0120499)
Supplement: S1 Table — (PDF) [file pone.0120499.s001.pdf]

S1 Table. Individual control data.

**Controls**										
Source		Code	Age	Gender	ASO Titer	D1Titer	D2Titer	Tubulin Titer	LysoGm1	CamK
Group 1	1		8	M	87	500	16000	1000	160	93
	2		13	M	221	1000	16000	1000	160	100
	3		9	F	166	2000	4000	500	80	99
	4		7	M	79	1000	16000	500	80	98
	6		8	M	271	500	2000	250	100	94
	8		11	M	70	500	2000	500	100	92
	10		9	F	78	1000	8000	1000	100	99
	11		10	M	240	1000	2000	500	80	88
	12		14	M	200	1000	4000	500	80	96
	13		12	M	200	1000	4000	500	80	94
	14		15	F	200	500	2000	250	160	86
	15		12	F	320	2000	8000	500	80	99
	16		15	M	25	1000	4000	1000	80	53
	17		15.5	F	25	500	4000	2000	NA	91
	18		13	M	35	2000	8000	2000	320	95
Group 2	1		17	M	63	1000	2000	500	80	79
	2		17	M	200	1000	4000	500	80	87
	3		7	F	25	2000	8000	1000	80	80
	4		12	F	25	4000	8000	1000	1280	72
	5		13	F	25	4000	8000	1000	320	88
	6		13	F	25	8000	4000	1000	320	90
	7		14	M	250	2000	16000	500	320	112
	8		10	F	25	8000	4000	500	160	80
	9		16	M	NA	4000	4000	500	640	82
	10		14	M	50	2000	4000	500	160	100
	11		16	F	160	2000	2000	1000	160	92
	12		9	M	125	1000	1000	500	80	104
	13		16	M	63	2000	2000	500	160	NA
	14		16	F	50	2000	8000	500	160	NA
	15		11	F	100	2000	8000	500	80	NA
	16		11	M	NA	2000	4000	500	80	NA
	17		10	M	250	1000	2000	500	80	NA
Group 3	2	66	7	M	95	2000	2000	500	320	NA
	3	88	13	F	84	2000	2000	1000	640	NA
	5	1	6	F	231	1000	4000	250	80	NA
	6	4	10	F	260	4000	4000	500	160	NA
	7	5	10	M	285	4000	8000	2000	320	NA
	10	14	13	M	241	500	500	500	320	NA

	11	64	14	F	188	8000	4000	1000	80	NA
	13	86	8	F	11	8000	4000	4000	640	NA
	16	96	5.5	F	258	4000	8000	500	320	NA
	17	97	14	F	64	1000	8000	500	160	NA
	18	61	5	F	24	8000	8000	500	640	NA
	19	73	2	M	134	1000	4000	250	320	NA
	20	75	9	M	100	2000	8000	500	640	NA
	21	80	13	M	335	2000	8000	1000	320	NA
	22	76	19	M	151	4000	32000	500	1280	NA
	24	78	7	F	276	2000	32000	1000	320	NA
	25	79	16	F	311	2000	32000	1000	640	NA
Group 4	1	1	7	F	55	1000	4000	1000	160	91
	2	2	8	F	57	2000	4000	4000	80	97
	3	7	6	M	47	1000	1000	1000	640	104
	4	9	6	M	36	1000	2000	1000	320	146
	5	11	6	F	25	2000	8000	2000	160	163
	6	13	5	M	25	8000	8000	2000	320	89
	7	14	8	F	164	2000	4000	1000	1280	83
	8	16	8	F	25	16000	32000	4000	640	130
	9	18	15	M	150	16000	8000	1000	160	184
	11	21	6	M	25	4000	16000	1000	1280	155
	12	22	8	M	25	2000	4000	1000	160	NA
	13	39	10	M	25	4000	8000	4000	640	NA
	14	41	8	F	NA	8000	8000	2000	640	NA
	15	117	14	F	174	2000	4000	1000	640	NA
	16	120	11	F	25	2000	4000	2000	1280	NA
	17	121	11	M	118	2000	2000	1000	1280	NA
	18	122	14	F	370	4000	4000	2000	1280	NA
	20	124	9	F	NA	16000	8000	2000	640	NA
	21	125	7	M	NA	2000	4000	1000	1280	NA
	22	126	11	M	NA	2000	4000	1000	1280	NA
	23	127	9	M	NA	4000	8000	2000	1280	NA
