# Supplementary material for: Neuronal Antibody Biomarkers for Sydenham’s Chorea Identify a New Group of Children with Chronic Recurrent Episodic Acute Exacerbations of Tic and Obsessive Compulsive Symptoms Following a Streptococcal Infection
Source: PLoS One. 2015 Mar 20;10(3):e0120499. doi: 10.1371/journal.pone.0120499 (PMC4368605; doi:10.1371/journal.pone.0120499)
Supplement: S2 Table — (PDF) [file pone.0120499.s002.pdf]

S2 Table. Anti-streptococcal ASO and anti-DNase B antibodies in longitudinal PANDAS-tics and OCD subjects.

a) Subjects with an exacerbation associated with a streptococcal infection (ExWS)

	Subject	Pre-Exac 1	Pre-Exac 2	Exac	Post-Exac 1	Post-Exac 2
ASO titer	1	40	40	160	160	120
	2	240	480	400	400	400
	3	100	80	80	60	60
	4	200	160	160	160	160
	5	25	25	25	120	100
	6	40	40	160	160	120
	Mean ± SD	108 ± 92	138 ± 175	164 ± 128	177 ± 116	160 ± 122
Anti-DNase B	1	120	120	960	1280	1280
	2	480	960	960	960	640
	3	1920	1600	1600	1600	960
	4	80	60	80	100	100
	5	25	25	25	25	25
	6	120	120	960	1280	1280
	Mean ± SD	458 ± 734	481 ± 652	764 ± 605	874 ± 661	714 ± 558

b) Subjects with an exacerbation without an associated streptococcal infection (ExWOS)

	Subject	Pre-Exac 1	Pre-Exac 2	Exac	Post-Exac 1	Post-Exac 2
ASO titer	7	60	50	60	60	60
	8	80	80	50	50	50
	Mean	70	65	55	55	55
Anti-DNAse B	7	60	60	60	60	60
	8	400	240	240	240	200
	Mean	230	150	150	150	140

ASO (antistreptolysin O); Exac (exacerbation).
